# Exploring the presence of markers of decidualization in the fallopian tubes: a systematic review

**DOI:** 10.1093/biolre/ioad062

**Published:** 2023-06-02

**Authors:** F Aljassim, N Georgopoulou, C H Rigby, S G Powell, J N R Wyatt, D K Hapangama, C J Hill

**Affiliations:** Department of Women’s and Children’s Health, Institute of Life Course and Medical Sciences, University of Liverpool, member of Liverpool Health Partners, Liverpool, UK; Department of Women’s and Children’s Health, Institute of Life Course and Medical Sciences, University of Liverpool, member of Liverpool Health Partners, Liverpool, UK; Department of Women’s and Children’s Health, Institute of Life Course and Medical Sciences, University of Liverpool, member of Liverpool Health Partners, Liverpool, UK; Department of Women’s and Children’s Health, Institute of Life Course and Medical Sciences, University of Liverpool, member of Liverpool Health Partners, Liverpool, UK; Department of Women’s and Children’s Health, Institute of Life Course and Medical Sciences, University of Liverpool, member of Liverpool Health Partners, Liverpool, UK; Department of Women’s and Children’s Health, Institute of Life Course and Medical Sciences, University of Liverpool, member of Liverpool Health Partners, Liverpool, UK; Liverpool Women’s Hospital NHS Foundation Trust, Member of Liverpool Health Partners, Liverpool, UK; Department of Women’s and Children’s Health, Institute of Life Course and Medical Sciences, University of Liverpool, member of Liverpool Health Partners, Liverpool, UK

**Keywords:** fallopian tubes, decidualization, implantation, ectopic pregnancy

## Abstract

The fallopian tubes (FTs) are part of the female upper genital tract. The healthy FT provides the biological environment for successful fertilization and facilitates the subsequent movement of the conceptus to the endometrial cavity. However, when the FT is damaged, as with salpingitis, pyosalpinx, and hydrosalpinx, it may increase the risk of an ectopic pregnancy, a life-threatening condition. Decidualization refers to a multifactorial process by which the endometrium changes to permit blastocyst implantation. The decidualization reaction is vital for endometrial receptivity during the window of implantation. To date, no comprehensive review that collates evidence on decidualization in the human FT has been conducted. Therefore, the aim of this review is to compile the current evidence on cellular decidualization occurring in the healthy and pathological FT in women of reproductive age. A literature search was conducted using five databases and identified 746 articles, 24 of which were analyzed based on inclusion and exclusion criteria. The available evidence indicates that the FT are able to undergo decidual changes under specific circumstances; however, the exact mechanism by which this occurs is poorly understood. Further research is needed to elucidate the mechanism by which decidualization can occur in the FT.

## Teaser

Under certain circumstances, the fallopian tubes exhibit molecular changes consistent with decidualization.

## Graphical Abstract

**Graphical Abstract:**
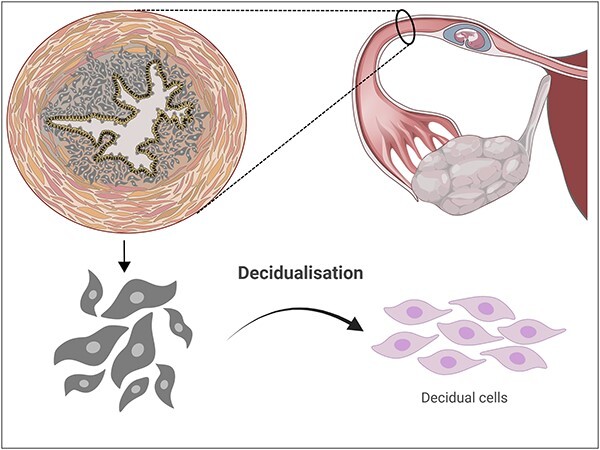

## Introduction

The fallopian tubes (FTs) are part of the female upper genital tract. In health, the FTs provide the biological environment for successful fertilization and facilitate the transport of the conceptus from the distal part of the FT to the endometrium [1]. However, pathological processes that cause tubal damage increase the chance of ectopic pregnancy (EP), a life-threatening condition [2]. EP is defined as the implantation of a blastocyst outside the endometrial lining of the uterus [3]. While EPs only occur in 2% of all pregnancies, they account for 8–9% of maternal mortality; over 95% of EPs are located in the FT, with the majority implanting in the ampulla [3, 4]. Intriguingly, tubal EP (tEP) appears to be restricted to primates and does not occur in other mammals. This distinction may be due to differences in uterine and tubal anatomy in primates, which allow for mixing of luminal fluids and thus potentially promote a more permissive environment for implantation in the FT [5]. Risk factors for tEP include, but are not limited to, previous tubal surgery, existing tubal pathology, and infection of the genital tract [6]. However, many EPs occur in women without any known risk factors [3]. In non-idiopathic tEP cases, the conventional postulation that ectopic implantation is a direct consequence of tubal damage has not been fully confirmed by the available evidence [3].

Pelvic inflammatory disease (PID) is the infection of the upper genital tract, which may manifest as pathologies of the FT [7]. These include salpingitis, pyosalpinx, and hydrosalpinx [7, 8]. Salpingitis refers to inflammatory and edematous FTs after ascending infection [7]. Pyosalpinx refers to a FT that is distended with pus due to obstruction following infection, inflammation, and subsequent formation of adhesions around the FT [7]. Hydrosalpinx describes a distended, fluid-filled FT that occurs as a result of tubal obstruction [7].

**Figure 1:**
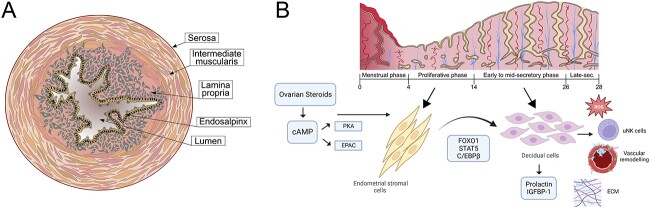
(A) Fallopian tube cross-section with major anatomical structures labeled. (B) Schematic representation of the decidualization process in human endometrium, which leads to enhanced reactive oxygen species (ROS) production, increased extracellular matrix (ECM) deposition, uterine natural killer cell (uNK) recruitment, and vascular remodeling. Abbreviations: CCAAT/enhancer-binding protein β (C/EBPβ), cyclic adenosine monophosphate (cAMP), exchange protein directly activated by cAMP (EPAC), forkhead box protein 1 (FOXO1), insulin-like growth factor binding protein-1 (IGFBP-1), protein kinase A (PKA), and signal transducer and activator of transcription 5 (STAT5). Created in part with BioRender.com.

The tubal mucosa, termed the endosalpinx, possesses a distinct profile of hormone receptor expression across the menstrual cycle, yet does not demonstrate the same dynamic changes in proliferative activity in response to hormones as the eutopic endometrium (Figure 1A) [9]. Normal embryo implantation occurs in the endometrium, and decidualization is considered a prerequisite for establishing a pregnancy [10]. A 2010 review exploring possible functional mechanisms by which risk factors predispose a tEP concluded that such molecular pathways have yet to be fully elucidated [10].

Decidualization, otherwise known as the decidual reaction, refers to a multifactorial process through which the endometrial stratum functionalis changes to allow a blastocyst to interact with the endometrium and implant. Decidualization includes both morphological and functional changes, of which the two most important are the differentiation of endometrial stromal cells to decidual cells, and leukocyte recruitment [11, 12]. Decidual transformation of stromal cells is primarily mediated by progesterone, which promotes intracellular accumulation of cyclic adenosine monophosphate (cAMP) [12]. Progesterone and cAMP regulate a network of signaling pathways; cAMP-mediated protein kinase A (PKA) is critical for decidualization, and exchange protein directly activated by cAMP (EPAC) can potentiate this process [13]. Downstream regulators of progesterone/cAMP signaling include forkhead box O1 (FOXO1), signal transducers and activators of transcription (STAT5), and CCAAT/enhancer-binding protein β (C/EBPβ). Decidual cells secrete factors that regulate embryo implantation and placentation, including insulin-like growth factor binding protein-1 (IGFBP-1) and prolactin (PRL) [11, 12]. Leukocytes play vital roles in decidual remodeling and immune tolerance of the endometrium during pregnancy establishment (Figure 1B) [14]. Humans are among the few viviparous species in which the endometrium will begin the process of decidualization during the post-ovulatory secretory phase of the menstrual cycle, independent of the presence of a conceptus [11, 12]. The decidual reaction is key to the endometrium being receptive, thus sanctioning the window of implantation, the timeframe within which the blastocyst can attach to and invade the superficial uterine wall [15]. An abnormal decidual response can lead to aberrations in placentation and, thus, both early and late gestational problems, such as recurrent implantation failure and preeclampsia [16].

To date, no comprehensive review has explored the available evidence on decidualization in the FT. Here, we conduct a systematic review to explore the potential for a decidual response in healthy and diseased FTs.

## Methods

This systematic review was reported in accordance with the Preferred Reporting Items for Systematic Reviews and Meta-Analysis (PRISMA) statement [17] and was preceded by a prospectively written protocol registered with PROSPERO (registration number: CRD42022333468) [18].

### Search strategy and selection criteria

A comprehensive literature search was conducted on 29 September 2022. Scopus, PubMed, CINAHL, EMBASE and EMCARE were searched for relevant published material. The search strategy included the following Medical Subject Heading (MeSH) terms, keywords, and their combinations: ("Fallopian tube" OR "Oviduct" OR "Uterine tube") AND ("Decidua"). No filters were applied to the search, and wildcards were incorporated to encompass various word endings where appropriate. All search results were uploaded into Rayyan [18], an electronic systematic review software enabling enhanced title and abstract screening. Duplicated literature was removed, and two independent reviewers performed a title and abstract screen according to the inclusion and exclusion criteria. Studies that met the following criteria were included: (1) concerning the decidualization of the human FT in health or benign pathology, (2) population of pre-menopausal or pregnant women, (3) publications in the English language. The exclusion criteria included (1) exclusive focus on malignant pathology; (2) animal studies; and (3) secondary, non-electronic, and gray literature. Following screening, full-text reviews were conducted by two independent reviewers, and a third reviewer was recruited for the resolution of any disagreements.

### Data extraction and analysis

Data from all eligible studies were extracted and recorded into an Excel spreadsheet recording the following: author, year of publication, study aim, sample size, comparator groups, experimental technique, relevant results, and author conclusions. Given the heterogeneity of both the methods and results of included studies, statistical meta-analysis was not feasible. Therefore, data have been presented thematically.

### Quality assessment

Risk-of-bias assessment was conducted by two independent reviewers (FA and CHR) using two well-established scoring tools. The Newcastle–Ottawa Scale (NOS) [19] was used for case–control and cohort studies and evaluated each study based on three domains: selection, comparability, and outcome. Each study receives a score between 0 and 9, which categorizes as either good, fair, or poor. In addition, a modified version of the NOS proposed by Murad et al. [20] was used for case series, which consists of eight questions across four domains: selection, ascertainment, causality, and reporting. Although a score between 0 and 8 can be attributed to each study based on binary responses to each question, Murad et al. suggest that numerical representation of methodological quality is not always recommended when certain questions are deemed more essential than others. Therefore, in this study, a judgment of methodological quality for each paper was made based on questions 1, 2, 3, 4, 6, and 8. The risk-of-bias assessment is detailed in Table 1 and Table 2.

**Table 1:** Risk-of-bias assessment for case–control and cohort studies using the Newcastle–Ottawa Scale

Author	Year	Selection				Comparability		Outcome			Total	
		1	2	3	4	5	6	7	8	9		
Case–control studies												
Al-Azemi	2009	★		★	★	★	★	★	★	★	8	Good
Basta	2010	★	★	★	★	★	★	★	★	★	9	Good
Inan	2004			★		★		★	★	★	5	Poor
Ji	2013	★		★	★	★	★	★	★	★	8	Good
Kuroda	2004			★		★			★	★	4	Poor
Pröll	2000	★		★		★		★	★	★	6	Fair
Refaat	2008			★	★	★		★	★	★	6	Fair
Refaat	2011	★	★	★	★	★	★	★	★	★	9	Good
Rutanen	1991	★				★			★	★	4	Poor
Von Rango	2001	★		★		★	★	★	★	★	7	Fair
Zygmunt	2000			★		★			★	★	4	Poor
Cohort studies												
Floridon	1999			★		★		★	★	★	5	Poor
Floridon	2000			★		★		★	★	★	5	Poor
Goffin	2003	★		★		★		★	★	★	6	Fair
Heatley	1996					★		★	★	★	4	Poor
Ordi	2006		★	★		★		★	★	★	6	Fair
Vassiliadou	1998	★			★	★		★	★	★	6	Fair

**Table 2:** Risk-of-bias assessment for case series using the tool proposed by Murad et al. (2018)

Author	Year	Selection	Ascertainment		Causality				Reporting	Total	
		1	2	3	4	5	6	7	8		
Hunt	2002	★	★	★		–		–		3	Fair
Land	1992		★	★	★	–		–		3	Fair
Randall	1987					–	★	–		0	Poor
Rewell	1971			★		–	★	–	★	0	Poor
Spornitz	1993			★		–	★	–	★	3	Fair
Tilden	1943	★				–	★	–	★	2	Poor
Wist	1954					–	★	–		1	Poor

## Results

**Figure 2:**
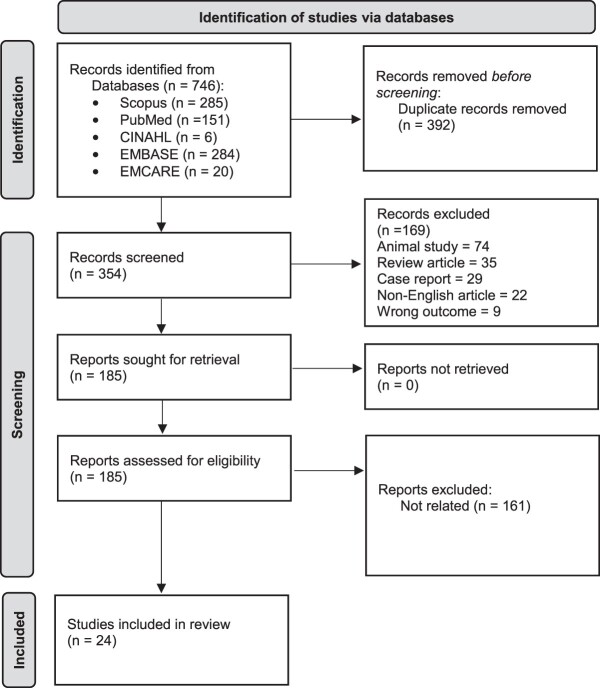
PRISMA flow diagram.

The literature search identified 746 unique articles; 354 remained after removing duplicate studies. Eligibility screening of these publications based on the assessment of their title and abstract, following the predetermined inclusion and exclusion criteria, led to the exclusion of a further 169 publications. The remaining 185 full-text articles were sought for retrieval, where, following evaluation, an additional 161 articles were excluded. Subsequently, 24 studies are included in the present review. This selection process is illustrated by a PRISMA flow diagram in Figure 2. Table 3 provides a summary of all studies included in this systematic review.

**Table 3:** Summary of studies that investigated decidualization in human fallopian tubes

First author	Title	Method(s)	Decidualization markers studied	Control/comparator group(s)	Relevant results
Al-Azemi et al. (2009)	The expression of MUC1 in human fallopian tube during the menstrual cycle and in ectopic pregnancy	IHC and quantitative RT-PCR	Mucin-1 (MUC1)	FT collected in the menstrual, follicular, or secretory phase following total abdominal hysterectomy for benign disease not affecting the tubes	Cyclical changes in MUC1 expression in tubal epithelial cells. Decrease in MUC1 mRNA and protein in tEP compared with pseudopregnancy
Basta et al. (2010)	The frequency of CD25^+^CD4^+^ and FOXP3^+^ regulatory T cells in ectopic endometrium and ectopic decidua	Fluorescence-activated single cell sorting	CD4, CD25, and forkhead box P3 (FOXP3)	Eutopic endometrium group derived from participants with regular menstrual cycles, and ectopic endometrium group derived from participants undergoing removal of ovarian endometriomas	The percentages of FOXP3^+^ cells in the subpopulation of CD4^+^ T lymphocytes found in the decidua of the patients treated for FT pregnancy were statistically significantly lower than both those observed in the ovarian endometriosis samples and those found in the secretory eutopic endometrium of the control group
Floridon et al. (1999)	Localization and significance of urokinase plasminogen activator and its receptor in placental tissue from intrauterine, ectopic and molar pregnancies	IHC	Urokinase plasminogen activator receptor (uPAR), urokinase plasminogen activator (uPA), cytokeratin and Ki67	Decidual tissue from normal intrauterine pregnancies, decidual tissue from complete and partial molar pregnancies, and pseudodecidual intrauterine tissue from participants with tEP	There was no decidual-like reaction of the stromal cells in the FT wall except in two cases with localized endometriosis. Only very few submucosal stromal cells away from the implantation site were positive for uPAR. The tubal epithelium, circumferential muscular cells, and serosal mesothelium were uPAR negative. No maternal stromal zone of uPAR-positive cells directed against the implanting ectopic pregnancy
Floridon et al. (2000)	Does plasminogen activator inhibitor-1 (PAI-1) control trophoblast invasion? A study of fetal and maternal tissue in intrauterine, tubal and molar pregnancies	IHC	Plasminogen activator inhibitor-1 (PAI-1)	Decidual tissue from normal intrauterine pregnancies, decidual tissue from complete and partial molar pregnancies, pseudodecidual intrauterine tissue from participants with tEP, and normal endometrial tissue from non-pregnant participants in the proliferative or secretory phase	In the tubal wall, PAI-1 was exclusively seen in a few submucosal stromal cells distant from the implantation site. The tubal epithelium and the muscular cells were PAI-1 negative. Decidualization of the stromal cells at the implantation site was not present
Goffin et al. (2003)	Evidence of a limited contribution of feto-maternal interactions to trophoblast differentiation along the invasive pathway	IF and quantitative RT-PCR	Connexin, cytokeratin, C-erbB-2, E-cadherin, epidermal growth factor receptor (EGFR), human leukocyte antigen G (HLA-G), human prolactin (hPRL), insulin-like growth factor binding protein 1 (IGFBP-1), integrins (α1, α6, α5β1), Ki67, p16, p57, and vimentin	First-trimester placental tissue	The decidualization markers PRL and IGFBP-1 were not detected in the tubal implantation sites
Heatley et al. (1996)	The immunophenotype of human decidua and extra-uterine decidual reactions	IHC	α_1_-Anti-trypsin, α_1_-anti-chymotrypsin, (human chorionic gonadotrophin β) β-hCG, CD3, CD20, CD45, CD68, cytokeratin, desmin, placental alkaline phosphatase (PLAP), smooth muscle actin, and S-100 protein	Decidua from intrauterine gestations, appendix, cervix and FT, and non-pregnant endometrium	Extrauterine mesenchymal cells, such as in the FT, that have undergone a decidual reaction correspond closely to their counterparts in the endometrial stroma. Post-partum decidualized stromal reaction was noted in the FT
Hunt & Lynn (2002)	Histologic features of surgically removed fallopian tubes	Microscopy	Histologic examination	None	Tubal ectopic decidua was found in 3% of the FT examined and were all from post-partum patients
Inan et al. (2002)	Immunolocalization of integrins and fibronectin in tubal pregnancy	IHC	Integrins (α3, αV, β1, and α2β1) and fibronectin	Non-pregnant FT samples and non-implantation site regions from tEP specimens	Integrins (in particular α3 and β1) and fibronectin may play a role in progression of tubal implantation. α3 and β1 integrins and fibronectin were present in ectopic pregnancy decidual cells at the site of implantation. Increased fibronectin-staining intensity may be related to the adhesive activity in tEP
Ji et al. (2013)	Reduced expression of aquaporin 9 in tubal ectopic pregnancy	IHC	Aquaporin 9 (AQP9), estrogen receptor (ER), and progesterone receptor (PR)	Non-pregnant FT samples collected during the mid-secretory phase, and non-implantation site regions from tEP specimens	Expression of AQP9 in the human FT may be significant during tubal implantation. No correlation between AQP9 and ER or PR in the non-implantation site or the normal FT. ER and PR had weak immunostaining at the site of implantation in tEP compared with spatially remote regions of the same tube
Kuroda et al. (2004)	The disappearance of CD34-positive and alpha-smooth muscle actin-positive stromal cells associated with human intra-uterine and tubal pregnancies	IHC	α-Smooth muscle actin (αSMA) and CD34	Non-pregnant FT, normal endometrium and intrauterine pregnancy decidual tissue	Cells positive for both antigens seemed to be more abundant in normal mucosa of FT than at the peri-decidual mucosa of intrauterine and tubal pregnancies. Neither αSMA^+^ nor CD34^+^ stromal cells were observed anywhere in the decidual stroma of intrauterine and tubal pregnancies, which may be an indicator of decidualization-induced changes in the stroma
Land & Arends (1992)	Immunohistochemical analysis of estrogen and progesterone receptors in fallopian tubes during ectopic pregnancy	IHC	Estrogen receptor (ER) and progesterone receptor (PR)	None	A decidual reaction was observed in 42% of tubal pregnancies, although the degree and extent of decidualization was less than normally seen in an intrauterine pregnancy. ER expression, PR expression, and serum β-hCG concentrations had no correlation to the degree of decidualization. The lack of PR in the FT may explain the absence of adequate decidualization in tubal pregnancies
Ordi et al. (2006)	Uterine (CD56^+^) natural killer cells recruitment: association with decidual reaction rather than embryo implantation	IHC	CD3, CD4, CD8, CD16, CD20, CD56, CD57, CD68, cytokeratin, and α-inhibin	Decidualized endometrium from participants undergoing progestin therapy, intrauterine pregnancy-associated ectopic decidua, and intrauterine decidua from spontaneous abortions	The immunomodulation of uterine natural killer (uNK) cells is most likely not induced by the local presence of trophoblast but is primarily hormonally regulated. No decidual reaction was detected in any ectopic implantation. Lack of uNK cells in all cases of tubal pregnancy. Ectopic decidua that is associated with intrauterine pregnancy is characterized by the presence of uNK cells
Pröll et al. (2000)	Tubal versus uterine placentation: similar HLA-G expressing extravillous cytotrophoblast invasion but different maternal leukocyte recruitment	IHC	BY55, CD1a, CD1b, CD1c, CD4, CD8, CD14, CD16, CD20, CD25, CD56, CD83, CD94, human leukocyte antigen DR (HLA-DR), HLA-G, leukocyte immunoglobulin-like receptors 1 and 2 (LIR1 and LIR2), cytokeratin, and vimentin	Non-pregnant FT samples and intrauterine decidua from elective terminations	Pregnant tubes were characterized by the lack of NK cells and of cells expressing CD94 receptor specific for HLA-E, and a prominent increase of CD8^+^ T cells, dendritic cells, and macrophages
Randall et al. (1987)	Placentation in the fallopian tube	Microscopy	Histologic examination	None	No histological evidence of any decidual reaction in ~76% of tubal pregnancies examined
Refaat et al. (2008)	The expression of activin-βA- and -βB-subunits, follistatin, and activin type II receptors in fallopian tubes bearing an ectopic pregnancy	IHC and quantitative RT-PCR	Activin-βA, activin-βB, activin receptor types 2A and 2B (ActRIIA and ActRIIB), and follistatin	Pseudopregnant FT samples collected from participants undergoing hysterectomy who were injected with β-hCG prior to surgery	Increased activin-A expression by the FT epithelial cells may stimulate tubal decidualization and trophoblast invasion within the tube. An increase in activin-A expression by the FT epithelial cells may increase the production of nitrous oxide in a concentration-dependent manner, which will result in pathological relaxation of the tubal smooth muscles, failure of propulsion of the early embryo along the FT, and the development of ectopic pregnancy
Refaat and Ledger (2011)	The expression of activins, their type II receptors and follistatin in human fallopian tube during the menstrual cycle and in pseudo-pregnancy	IHC and quantitative RT-PCR	Activin-βA, activin-βB, ActRIIA, ActRIIB, β-actin, and follistatin	Non-pregnant FT samples from the proliferative, secretory, and menstrual phases, and pseudopregnant FT samples collected from participants undergoing hysterectomy who were injected with β-hCG prior to surgery	Exposure of the tubal epithelium to β-hCG modulates the expression of tubal activins which are involved in regulation of tubal physiology and early embryonic development
Rewell (1971)	Extra-uterine decidua	Microscopy	Histologic examination	None	Decidua was found in the FT in 3% of cases associated with intrauterine pregnancy before the 18th week of gestation or in the puerperium, and found in all contralateral tubes in tubal pregnancies
Rutanen et al. (1991)	Decidual transformation of human extrauterine mesenchymal cells is associated with the appearance of insulin-like growth factor-binding protein-1	IHC	IGFBP-1	Decidual tissue from early pregnancy	25% of the FT studied, all retrieved following tubal ligation, contained decidual cells, which were morphologically indistinguishable from those in endometrium. These cells stained positively for IGFBP-1, which the authors suggest proves IGFBP-1 involvement in decidual transformation
Spornitz (1993)	Pseudo-decidualization at the site of implantation in tubal pregnancy	Microscopy	Histologic examination	None	The cells present at the tubal implantation site are suggested to be named “pseudo decidual cells,” as apart from their large size, they do not possess the miniature mitochondria, basal lamina-like coat, or the decidual granules seen in decidual cells. In addition, they are not of maternal origin, thus it was indicated that ectopic endometrium is not involved in tubal implantation
Tilden and Winstedt (1943)	Decidual reactions in fallopian tubes: histologic study of tubal segments from 144 post-partum sterilizations	Microscopy	Histologic examination	None	12% of tubal segments retrieved following post-partum sterilizations exhibited decidual formation of varying extent and location. This study suggests that the receptivity of the tubal mucosa to the fertilized ovum may play a more important role in ectopic implantation than generally believed
Vassiliadou and Bulmer (1998)	Characterization of tubal and decidual leukocyte populations in ectopic pregnancy: evidence that endometrial granulated lymphocytes are absent from the tubal implantation site	IHC	CD3, CD20, CD43, CD45, CD45RA, CD56, CD57, and CD68	Non-implantation site regions from tEP specimens, and first-trimester intrauterine decidua from participants with tEP	Tubal decidualization was not observed in any of the specimens examined. Macrophages and T cells were the most abundant leukocyte populations at the tubal implantation site
Von Rango et al. (2001)	Effects of trophoblast invasion on the distribution of leukocytes in uterine and tubal implantation sites	IHC	Cytokeratin, CD8, CD20, CD45, CD56, and CD68	Decidual tissue obtained from elective terminations of normal intrauterine pregnancies, and intrauterine decidua from participants with tEP	Leukocyte populations present in the tubal and uterine mucosa are an intrinsic characteristic of these tissues. The number of CD45^+^ leukocytes, mainly composed of CD68^+^ macrophages, increase from non-pregnant FT to tEP; the distinct leukocyte distribution pattern at the implantation sites suggests that the invading trophoblast exerts a paracrine influence on endometrial and endosalpingeal leukocytes. The absence of natural killer cells from the tubal wall may be one reason for the higher degree of invasiveness of the trophoblast at the tubal implantation site
Wist (1954)	Decidual reaction and tubal pregnancy	Microscopy	Histologic examination	Non-implantation site regions from tEP specimens	Decidual reactions were observed in 17% of cases of tubal pregnancy, the majority of which appeared to occur away from the implantation site. This indicates that there is no chemotactic attraction between the ovum and decidual reaction
Zygmunt et al. (2000)	Local fetal signal is not required for maintaining IGFBP gene expression in the human decidua: evidence from extrauterine pregnancies	In situ hybridization and IHC	Insulin-like growth factor 2 (IGF-II), IGFBP-1, IGFBP-2, IGFBP-3, IGFBP-4, IGFBP-5, IGFBP-6, cytokeratin, and vimentin	Endometrial tissue obtained from participants with tEP, and intrauterine decidua and FT tissue from elective terminations of normal pregnancies	Abundant IGFBP-1 mRNA was present in the decidualized segments of the tubal wall in intrauterine pregnancies. Other IGFBP mRNAs were expressed in moderated abundance (IGFBP-3, IGFBP-4). These findings suggest that the expression of IGFBP-1 mRNA is equal to the hormonally induced differentiation of endometrial or tubal stromal cells into decidua, rather than to that induced locally by the conceptus

Abbreviations: FT (fallopian tube), IHC (immunohistochemistry), IF (immunofluorescence), RT-PCR (reverse transcription polymerase chain reaction), tEP (tubal ectopic pregnancy)

## Thematic analysis

### Decidualization associated with intrauterine pregnancy in post-partum fallopian tubes

While rare, decidual changes do occur in the FT, which is primarily evident from post-partum FT of intrauterine pregnancies (IUP) that show decidual changes. Ordi et al. (2006), Hunt and Lynn (2002), Rutanen et al. (1991), Tilden and Winstedt (1943), and Heatley et al. (1996) reported a decidual reaction in post-partum tubes associated with an IUP; Rewell (1971) reported tubal decidualization associated with IUP and in contralateral tubes of tEP [21–26]. Heatley et al. (1996), Rutanen et al. (1991), and Tilden and Winstedt (1943) examined FT samples collected from sterilization procedures performed at term pregnancies [23, 24, 26]. Rewell (1971) investigated the FT in the puerperal period [25]. However, Ordi et al. (2006) and Hunt and Lynn (2002) do not explicitly state the circumstances of sample collection nor period post-partum [21, 22]. Collectively, studies indicated that 3–25% of post-partum women demonstrate a tubal decidual reaction [23, 25]. However, the study by Rutanen et al. (1991), which concluded that 25% of tubes showed decidualization, only included eight samples compared to the 194 post-partum tubes analyzed by Rewell (1971), where only 3% had decidual changes [23, 25].

### Decidualization associated with tubal ectopic pregnancy

Decidualization associated with tEP has been described in several studies. Nine included papers observed a decidual reaction in the FT containing the tEP at the site of implantation, away from the site of implantation within the same tube, or at both sites [25, 27–35]. One study has also demonstrated decidualization in the contralateral FT in women with tEP [25]. However, Floridon et al. (1999) detected tubal decidualization only in two cases of tEP with localized endometriosis from a total of 50 tEP specimens. None of the above studies described the decidualization to be as extensive as would be expected at the implantation site in a normal IUP.

Ordi et al. (2006), Goffin et al. (2006), and Vassiliadou et al. (1998) analyzed a total of 41 tEP specimens and concluded an absence of a decidual reaction at the site of implantation [21, 36, 37]. Interestingly, Randall et al. (1987) demonstrated that cells, which initially resembled decidual cells at the site of implantation, were in fact of cytotrophoblastic origin [32].

### Leukocyte infiltration in the fallopian tube

A study by Von Rango et al. (2001) indicated that the number of CD45^+^ leukocytes increased in the tubal mucosa from non-pregnant to tEP and suggest it to be a consequence of increased numbers of CD68^+^ macrophages [38]. In tEP, there is a marked lack of CD56^+^ uterine natural killer (uNK) cells, which are thought to limit trophoblast invasion in normal IUPs [21, 37–39]. Ordi et al. (2006) found that increased recruitment of uNK cells in decidual tissue is a common phenomenon regardless of location and that this process is mediated by hormones rather than the presence of an implanting blastocyst [21]. In addition, Von Rango et al. (2001) stated that while uNK cells are not necessary for successful implantation, they may limit trophoblast invasion; thus, the absence of uNK cells in the FT is proposed to allow for the increased trophoblastic invasion seen in tEP [38].

The most abundant leukocytes identified in tEP were macrophages and T cells [37–39]. When comparing the leukocyte populations at the tEP implantation site with the matched intrauterine decidua, the numbers of T cells and macrophages were similar [39]. Basta et al. (2010) reported a significantly lower percentage of T regulatory cells in the subpopulation of CD4^+^ T lymphocytes in the decidual of tEP compared to the secretory phase eutopic endometrium [30].

### Cellular markers of decidualization

The studies included in this review investigated various cellular markers of decidualization. In particular, two studies by Refaat et al. (2008, 2011) employed immunohistochemistry and quantitative reverse transcription polymerase chain reaction to quantify the expression of activins in tEP. These studies suggest that activins have a paracrine and autocrine action in the FT; in the endometrium, decidualization is facilitated by activins increasing the expression of matrix metalloproteinases [40, 41]. The increased expression of activins in tEP, when compared to secretory phase tubes, is considered pathological. However, it is also suggestive of tubal decidualization because activins are raised in the cycling endometrium during the luteal phase [40, 41]. Refaat et al. (2008) also investigated the action of follistatin in tEP. Their findings indicated that the expression of activins and follistatin might play an important role in the pathogenesis of ectopic implantation but not necessarily in determining the site of implantation. The authors propose that increased expression of activin-A in the FT could increase nitric oxide production, which may induce a pathological relaxation in the smooth muscle of the FT. This muscular relaxation would prevent adequate movement of an embryo, which in turn could increase the chance of a tEP [40, 41]. This theory is supported by a similar finding, whereby the embryo was located in the same place as a decidual polyp in the tube; Wist et al. (1954) suggest that the tubal obstruction prevented the embryo from moving through the tube and therefore caused a tEP [33]. Wist et al. (1954) proposed that the decidual reaction should be considered a result of pregnancy but not the cause of the tEP [33].

The expression of mucin-1 (MUC1) in tubal epithelial cells fluctuates throughout the menstrual cycle [42]. In the luteal phase, increased MUC1 expression in tubal epithelial cells may act as a protective mechanism against ectopic implantation, which might include an anti-adhesive effect and/or facilitate transport [42]. In tEP, decreased MUC1 expression indicates feature changes in the tubal epithelium [42].

Fibronectin is a ligand for integrins that is present at the implantation site in the endometrium and has a key role in embryo implantation following its adhesion to the maternal tissue. Integrins and fibronectin, which are considered necessary for uterine implantation, have also been shown to be present in tEP, indicating that they may have a role in tubal implantation [27]. Kuroda et al. (2004) observed the total loss of alpha-smooth muscle actin (αSMA) and CD34^+^ stromal cells in both IUP and tEP compared to non-pregnant endometrial and tubal tissues. Loss of αSMA^+^ and CD34^+^ stromal cells may therefore indicate decidualization-specific changes in tEP [31].

A study by Ji et al. (2013) compared estrogen receptor (ER) and progesterone receptor (PR) expression between normal mid-secretory non-pregnant tubes with tEP, both at the site of implantation and at distant regions of the same tube. They reported a decrease in the expression of ER and PR at the site of implantation compared to other tubal regions of the pregnant FT and secretory phase non-pregnant tubes; the expression of ER and PR in the latter two groups was similar. Expression of ER and PR was mainly confined to the epithelial nuclei and sparsely in the tubal stroma [43]. Land and Arends (1992) suggest that the absence of sufficient decidualization in tubal pregnancies may be explained by the lack of PR in the FT [28], yet the action of progesterone via PR is known to reduce the expression level of its own receptor and ER [44].

Three included articles investigated bona fide decidual markers in the FT. Groffin et al. (2003) reported an absence of expression of two well-known decidualization markers, PRL and IGFBP-1, in tEP [36]. Rutanen et al. (1991) and Zygmunt et al. (2000) indicated IGFBP-1 expression in FT in post-partum and intrauterine pregnancies, respectively [23, 45].

## Discussion

The objective of this review was to compile the available evidence regarding the potential of the FT to undergo decidualization. We found that the FT has the ability to undergo stromal decidualization under specific circumstances [31, 45]. However, unlike the endometrium, where decidualization is a hallmark of the secretory phase, decidualization in the FT appears to be a relatively rare occurrence, and it is unclear how or why it transpires.

In the endometrium, decidualization is modulated by cyclic fluctuations in the ovarian steroid hormones, estrogen and progesterone [46]. In the absence of an embryo, the superficial endometrial layer is shed during menses. Endometrial decidua can be characterized by morphological changes, phenotypic markers, and a unique immune cell profile.

In the luteal phase, the rise in progesterone levels stimulates a chain of reactions in the endometrial stromal cells, causing an upregulation of multiple genes, including the classical markers of decidual cells, PRL and IGFBP-1 [11]. Endometrial receptivity also involves the presentation of adhesion molecules and simultaneous loss of inhibitory factors that prevent embryo attachment [47]. The phenotypical changes of the endometrium include vascular remodeling, an influx of uNK cells, and the differentiation of stromal cells to a hypertrophic, secretory phenotype [12, 48]. There is a fivefold increase in leukocytes during the secretory phase, of which the most notable change is the significant increase in uNK cells; uNK cells account for approximately 70% of the total leukocyte population [21, 47, 49].

As identified in this review, the FT have the ability to decidualize under specific circumstances. One explanation is that the FT is more sensitive to the higher concentrations of progesterone produced by the placenta compared to the relatively moderate levels produced by the corpus luteum, which is why decidual changes in the FT associated with IUP are present post-partum [25].

It is important to note that the hormone responsiveness of the tubal mucosa is proposed to be different to that of endometrial cells. The dynamic changes in the expression of steroid hormone receptors are not observed in healthy pre-menopausal tubes when compared with the eutopic endometrium [9]. The relative hormone resistance of the tubal mucosal would prevent initiation of decidualization, but in the event that the cells become sensitized, possibly via prolonged and sustained exposure to high progesterone levels, decidualization may occur and promote tEP.

Furthermore, there is a paucity of studies investigating the FT in early IUP to confirm that this is only a late pregnancy event. For obvious reasons, access to such material is limited. The developing fetus and placenta of ongoing IUPs produce many endocrine agents that may influence the tubal mucosa, potentially inducing decidual changes [11, 12, 14]. Intriguingly, no studies have investigated tubal changes following exogenous progestogen administration, which typically induces a decidualization response in the endometrium. Therefore, further studies are needed to conclude on the decidualization potential of healthy FT.

The receptive endometrium describes the stage at which an embryo can implant, and it can have degrees and types of abnormality [50]. In parallel, extravillous trophoblasts are thought to switch from a differentiating phenotype to an invasive phenotype, which is believed to occur independently of the maternal environment [36], meaning that the embryo could begin to invade any tissue that it is in contact with when this change occurs. This postulation is acceptable, considering the observation of rare ectopic pregnancies in the abdomen. Evidence suggests that tubal implantation may occur due to stagnation of the embryo in the FT; such immobility will allow prolonged exposure of the FT to the secretory products of an embryo, which may induce a local decidual reaction in the FT, encouraging tubal implantation [33]. However, stagnation of the embryo in the FT in the study by Wist et al. (1954) occurred due to the presence of a decidual polyp [33]. Interestingly, pseudoxanthomatous salpingitis manifests histological similarities to decidualization [51]. As the study by Wist et al. was published in 1954, it can be speculated that the multiple decidual polyps were, in fact, expanded plicae due to the presence of numerous histiocytes [33, 51].

Activins are important autocrine/paracrine regulators that stimulate and facilitate endometrial decidualization, which is crucial for successful implantation [52]. They are secreted by newly decidualized cells, promoting the spread of decidualization throughout the endometrium [40, 53–55]. The presence of specific molecular markers, such as activins in the FT, affects tubal mobility via altering smooth muscle contractility and/or ciliary beat activity, leading to tubal transport failure and, consequently, blastocyst retaining within the tube, which overexposed the tubal epithelium to the embryonic chorionic gonadotrophin, and ultimately induces tubal epithelial receptivity [9, 38–40].

Although the immune cell profile of the FT may have similarities to that of the endometrium, there are stark differences, such as the increased number of T cells and the lack of uNK cells in the FT. The absence of uNK cells in the FT may allow over-invasion of the extravillous trophoblasts [36], which could be a reason for frequent rupture of the FT observed in tEP. Unlike the endometrium, the immune cell profile of the FT does not appear to change in response to an embryo implanting. This is again likely to reflect the relative resistance of the tubal mucosa to steroid hormones [9]. Von Rango et al. (2001) identified T cells, followed by macrophages, as the most abundant leukocytes in the healthy FT [38]. This immune profile is similar to that of the proliferative phase endometrium described by Vallvé-Juanico et al. (2019), whereby the most abundant leukocytes are T cells, followed by macrophages and uNK cells [56], although uNK cells are virtually absent from tubal mucosa [38, 57].

In summary, there is insufficient evidence to define the decidualization potential of healthy and pathological FT in full. Furthermore, no studies have explored decidualization reactions in damaged FT, such as hydrosalpinx after infection. Therefore, it remains challenging to find a causal relationship between factors influencing tEP. This uncertainty creates a causality dilemma, in which it is difficult to confirm what came first: the embryo expressing the invasive phenotype or a pre-existing receptive FT. In addition, the association of ectopic pregnancy with many risk factors, such as previous FT surgery and PID, is well established; however, this review did not identify any literature exploring decidualization in such cohorts. Furthermore, there is a lack of studies regarding decidualization in the FT during specific stages of the menstrual cycle.

## Conclusions

The FT can undergo decidual changes under specific circumstances. These may include prolonged exposure to high levels of progesterone, placental products, and prolonged exposure to a conceptus. The presence of decidual cells in tEP is poorly understood, and many questions are left unanswered. Further research surrounding the decidualization of the FT at different stages of the menstrual cycle and following damage would help to bridge the gap of knowledge in understanding the pathophysiology of the FT. In addition, receptivity markers, the proliferation of the tubal mucosa, and the immune profile of normal and damaged tubes could be explored in FT across the menstrual cycle. Such studies could provide greater insight into the mechanisms of aberrant embryo implantation at ectopic sites.

## Authors’ roles

FA, NG, SGP, JNRW, DKH, and CJH developed the systematic review protocol. FA, NG, and CHR performed database searches and data extraction. FA and CJH created figures. NG, CHR, and FA wrote the first draft of the manuscript. All authors finalized, critically appraised, and approved the final version of the manuscript.

**Conflict of Interest**: The authors have declared that no conflict of interest exists.

## Data availability

The data underlying this article are available in the article.

## Notes

Grant Support: FA is supported by Imam Abdulrahman bin Faisal University, Saudi Arabia (30199). NG and CHR are supported by a University of Liverpool Master’s studentship. JNRW and SJP are supported by clinical fellowships at Liverpool University Hospitals NHS Foundation Trust. CJH is supported by a Wellbeing of Women project grant (RG2137), SRI/Bayer and Wellcome Trust IFFS3. DKH is supported by a Wellbeing of Women project grant (RG2137) and an MRC clinical research training fellowship (MR/V007238/1).
